# Supplementary material for: Conformational transitions of the Spindly adaptor underlie its interaction with Dynein and Dynactin
Source: J Cell Biol. 2022 Sep 15;221(11):e202206131. doi: 10.1083/jcb.202206131 (PMC9481740; doi:10.1083/jcb.202206131)
Supplement: Table S1 — shows crystallographic data. [file JCB_202206131_TableS1.docx]

**Table S1**

**Crystallographic data**

| Dataset | Spindly^1-100^ | SeMet-Spindly^1-100^ |  |
| --- | --- | --- | --- |
| Spacegroup | P 3_2_ 2 1 | P 1 2_1_ 1 |  |
| a=b, c (Å) | 112.51, 49.64 | 112.33, 48.65 |  |
| Wavelength (Å) | 0.9999 | 0.91587 |  |
| Resolution (Å) | 48.8 - 2.8 (2.95 - 2.80) | 97.3 - 3.4 (3.58 - 3.4) |  |
| R_merge_ | 0.079 (1.558) | 0.139 (0.769) |  |
| R_pim_ | 0.050 (0.970) | 0.026 (0.136) |  |
| I/σ(I) | 10.8 (1.5) | 15.0 (4.9) |  |
| CC_1/2_ | 0.99 (0.72) | 0.99 (0.99) |  |
| Completeness(%) | 99.3 (99.6) | 100 (100) |  |
| Redundancy | 5.4 (5.6) | 33.2 (33.0) |  |
| Refinement | | Structure determination (SAD) – CRANK2 | |
| Resolution (Å) | 2.8 | Phasing |  |
| Reflections (work, test) | 9068, 342 | CFOM substructure | 69.4 |
| R_work_ / R_free_ | 0.247 / 0.290 | Se sites used | 18 |
| No. atoms |  | FOM Phasing | 0.2031 |
| Protein (chain A, B) | 1570, 1589 | FOM Density Modification | 0.3764 |
| B factors (Å^2^) |  | Automated Model Building |  |
| Protein (chain A, B) | 75.1, 73.4 | R_free_ | 0.3287 |
| R.m.s deviations (RMSZ score) |  | Built Residues | 206 |
| Bond lengths | 0.0149 (0.564) | Built Fragments | 8 |
| Bond angles | 1.831 (0.908) | Docked Residues (%) | 72.8 |
| Favorable/Allowed/Outliers | 189/0 |  |  |
| Molprobity score (%ile) | 2.93 (97) |  |  |
