# Supplementary figures and images for: Conformational transitions of the Spindly adaptor underlie its interaction with Dynein and Dynactin
Source: J Cell Biol. 2022 Sep 15;221(11):e202206131. doi: 10.1083/jcb.202206131 (PMC9481740; doi:10.1083/jcb.202206131)

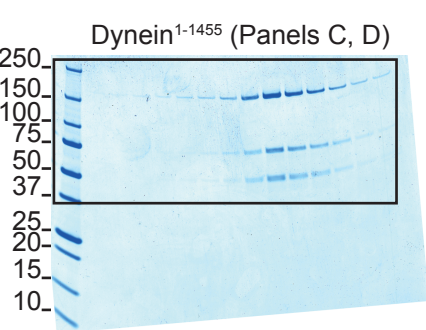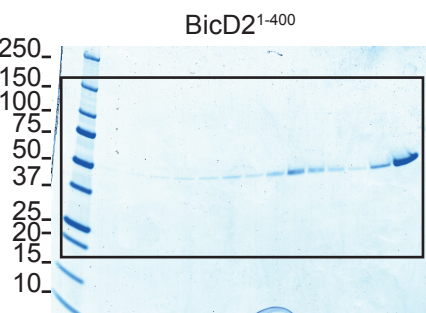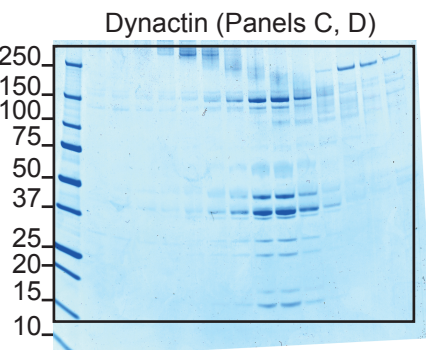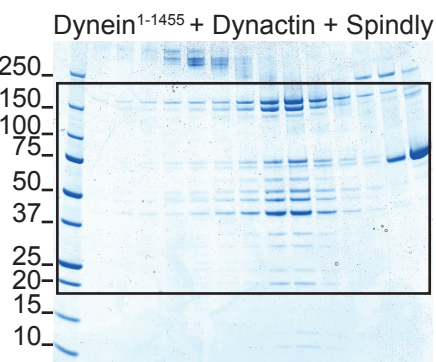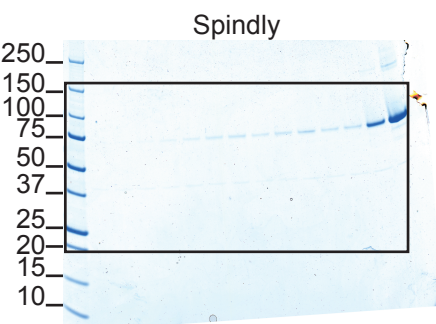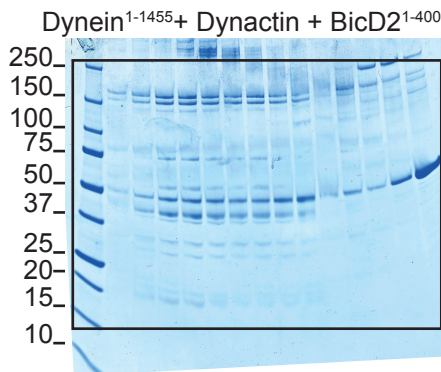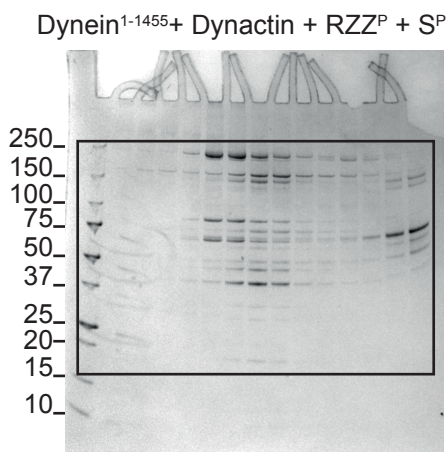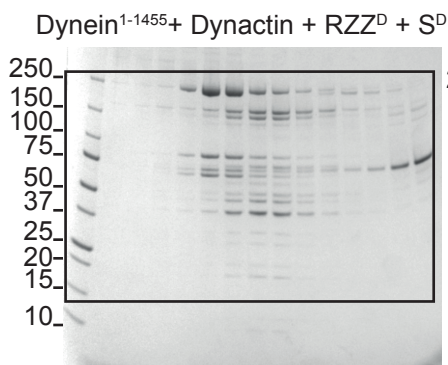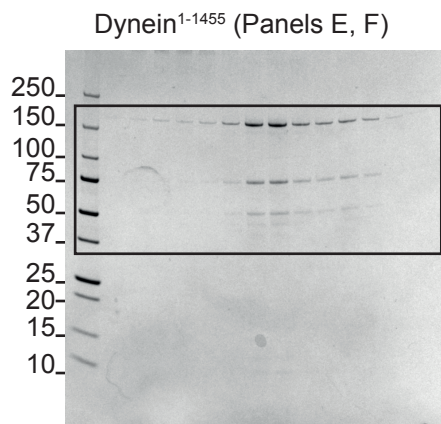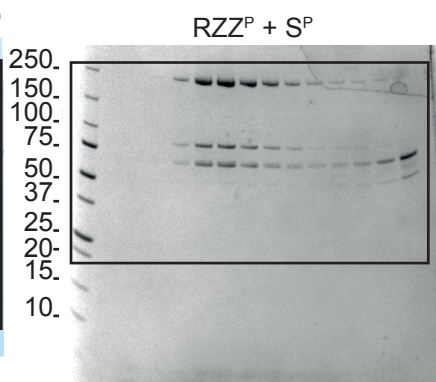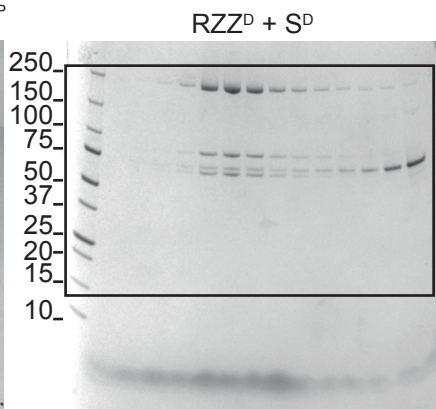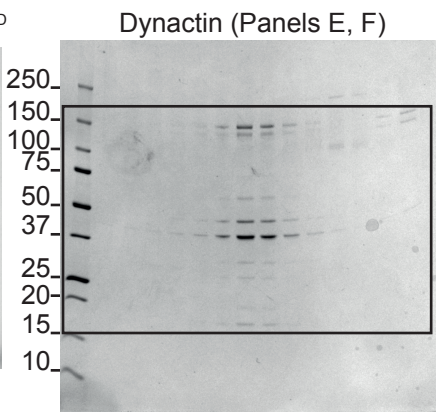

Supplement: SourceData F2 — contains original blots for Fig. 2. [file JCB_202206131_SourceDataF2.pdf]

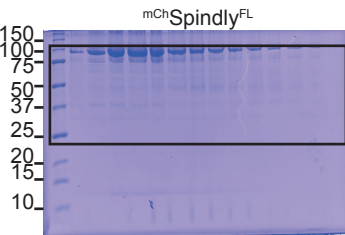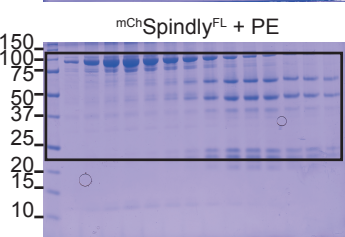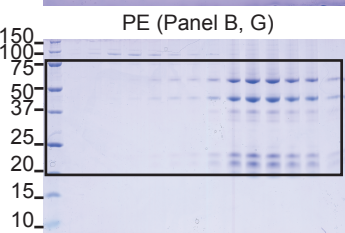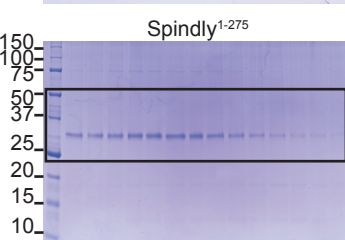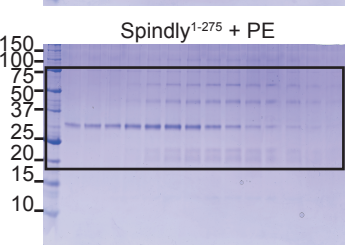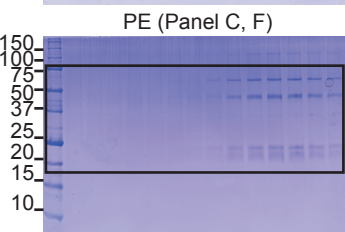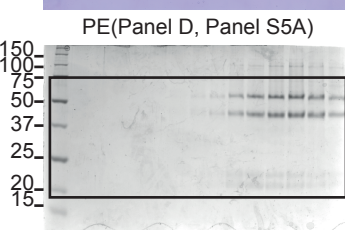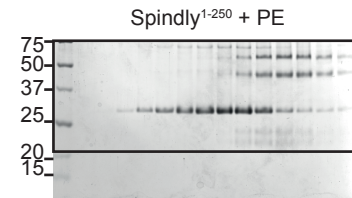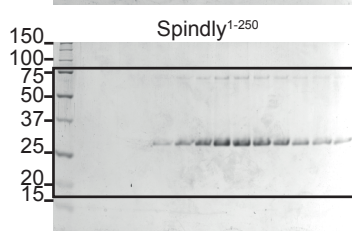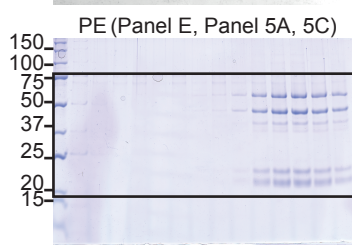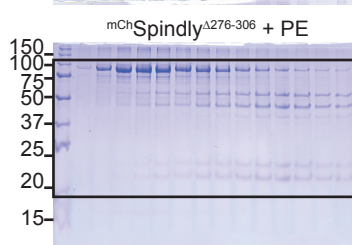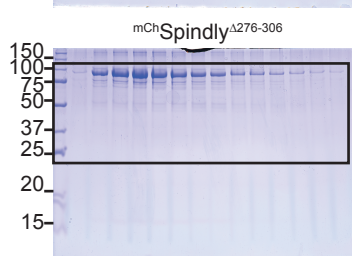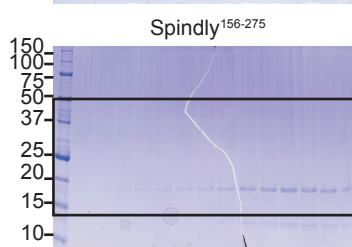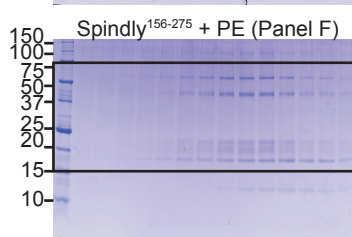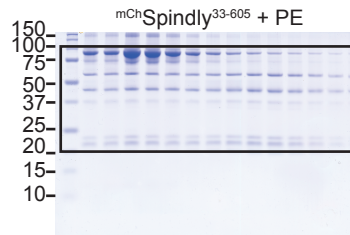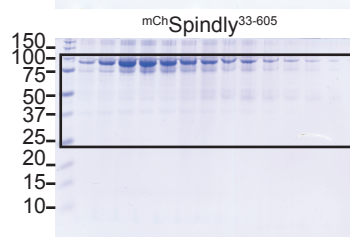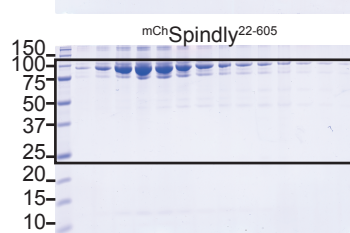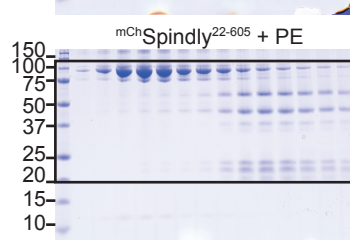

Supplement: SourceData F3 — contains original blots for Fig. 3. [file JCB_202206131_SourceDataF3.pdf]

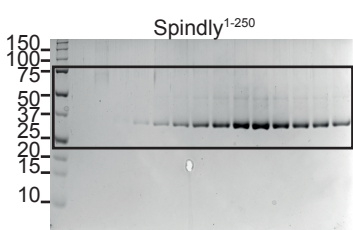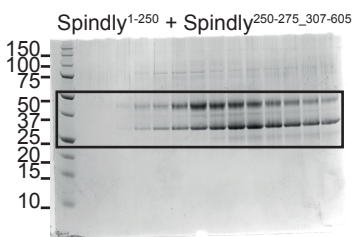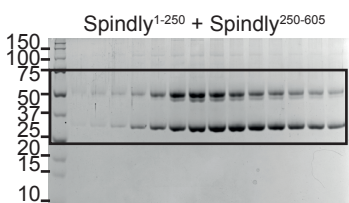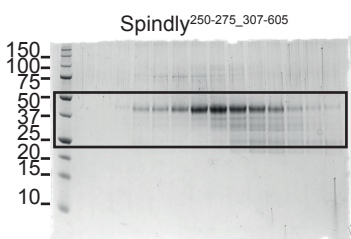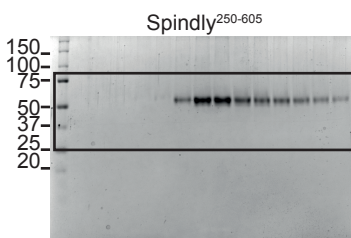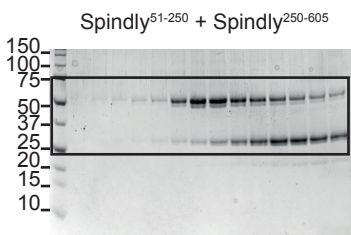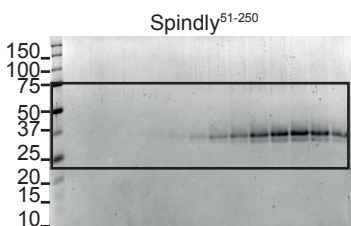

Supplement: SourceData F4 — contains original blots for Fig. 4. [file JCB_202206131_SourceDataF4.pdf]

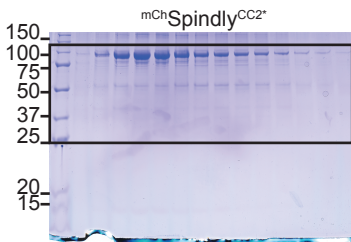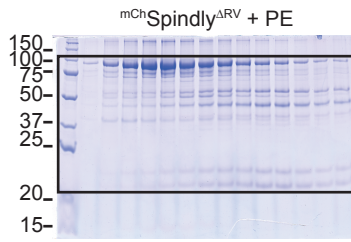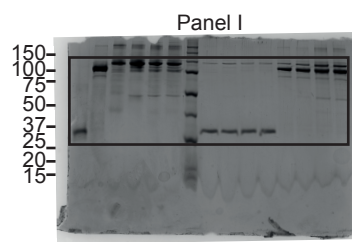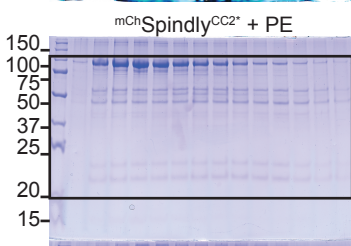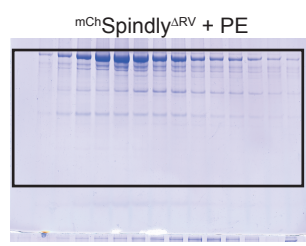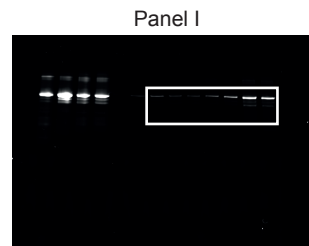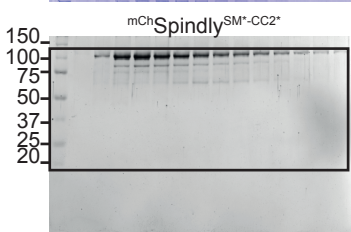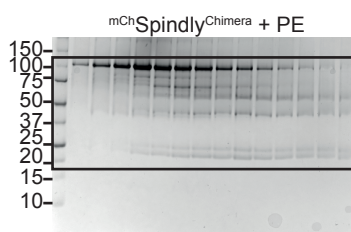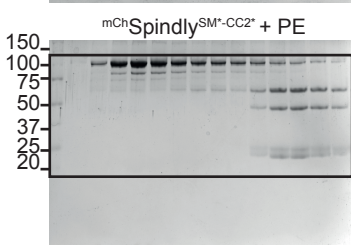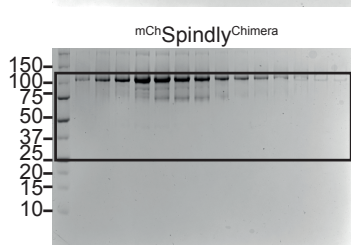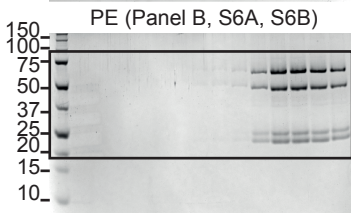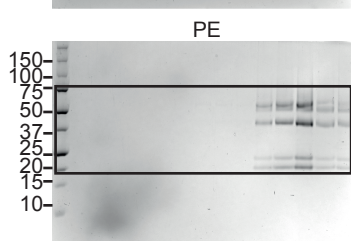

Supplement: SourceData F5 — contains original blots for Fig. 5. [file JCB_202206131_SourceDataF5.pdf]

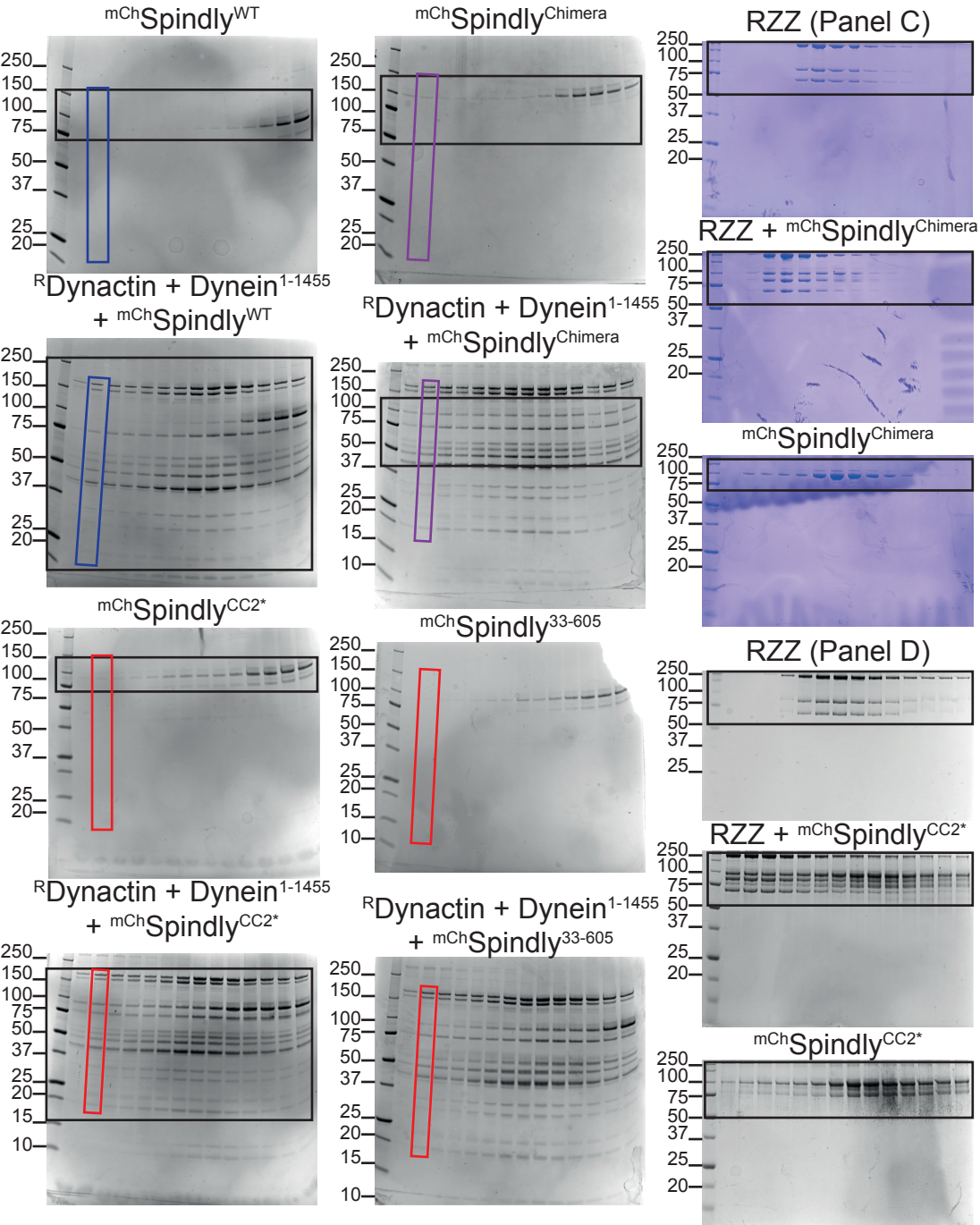

Supplement: SourceData F6 — contains original blots for Fig. 6. [file JCB_202206131_SourceDataF6.pdf]

Panel G Panel H

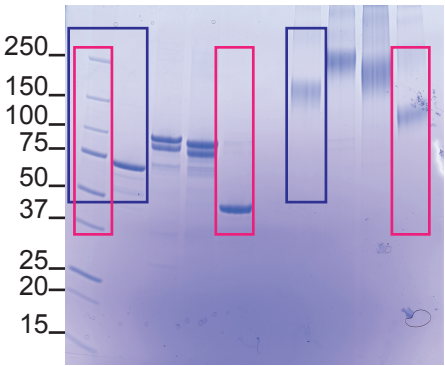

Panel J

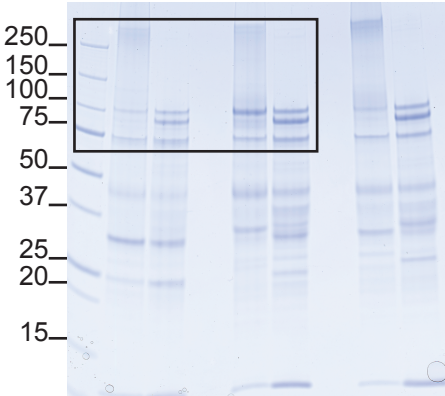

Panel K

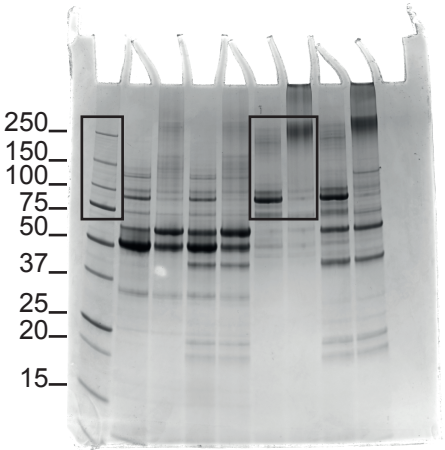

Supplement: SourceData FS1 — contains original blots for Fig. S1. [file JCB_202206131_SourceDataFS1.pdf]

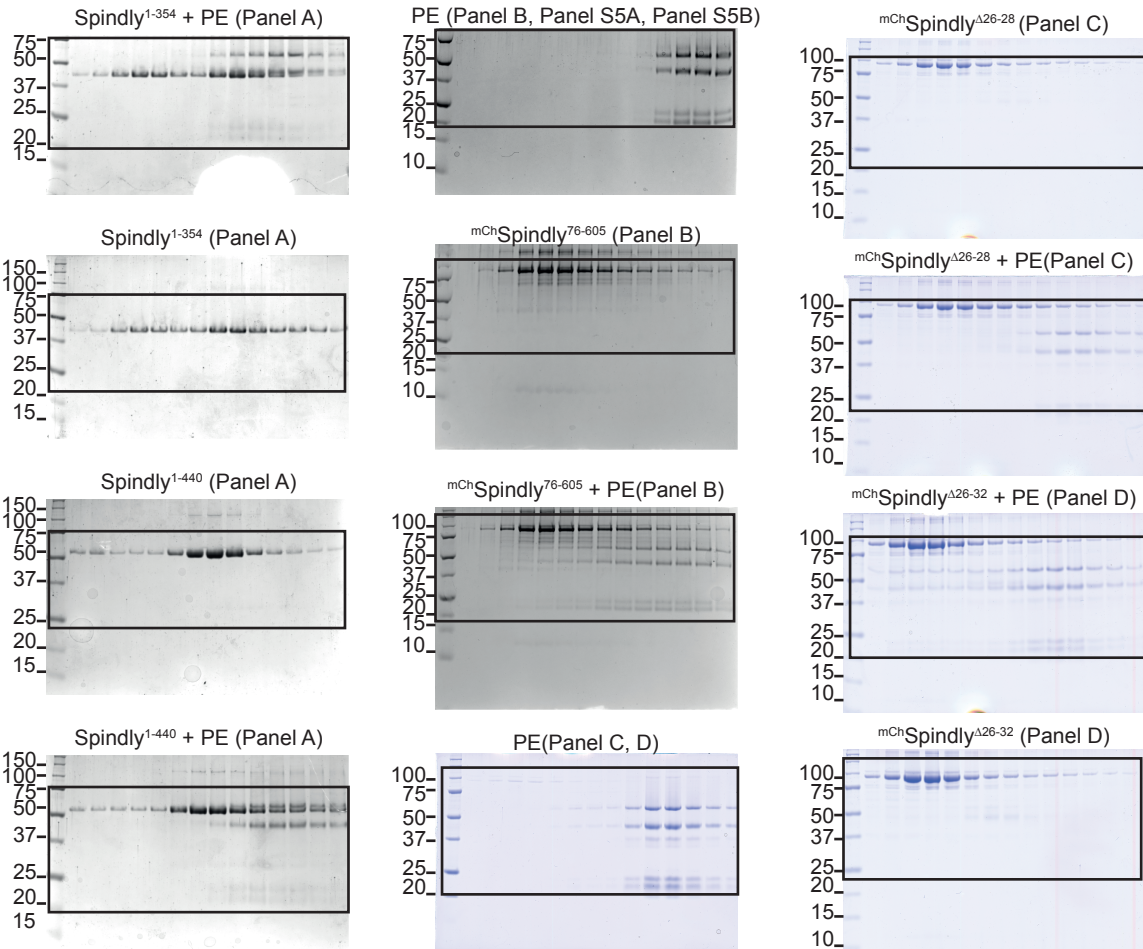

Supplement: SourceData FS5 — contains original blots for Fig. S5. [file JCB_202206131_SourceDataFS5.pdf]

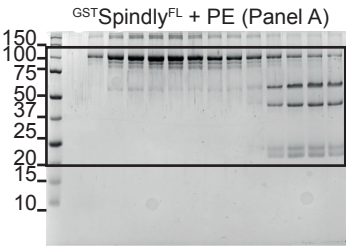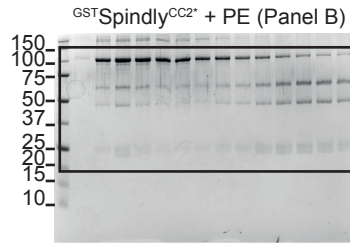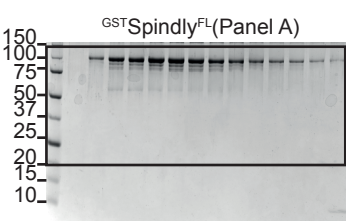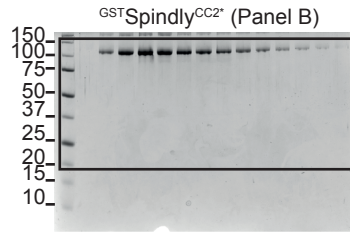

Supplement: SourceData FS6 — contains original blots for Fig. S6. [file JCB_202206131_SourceDataFS6.pdf]
