# Supplementary material for: Conformational transitions of the Spindly adaptor underlie its interaction with Dynein and Dynactin
Source: J Cell Biol. 2022 Sep 15;221(11):e202206131. doi: 10.1083/jcb.202206131 (PMC9481740; doi:10.1083/jcb.202206131)
Supplement: SourceData FS4 — contains original blots for Fig. S4. [file JCB_202206131_SourceDataFS4.pdf]

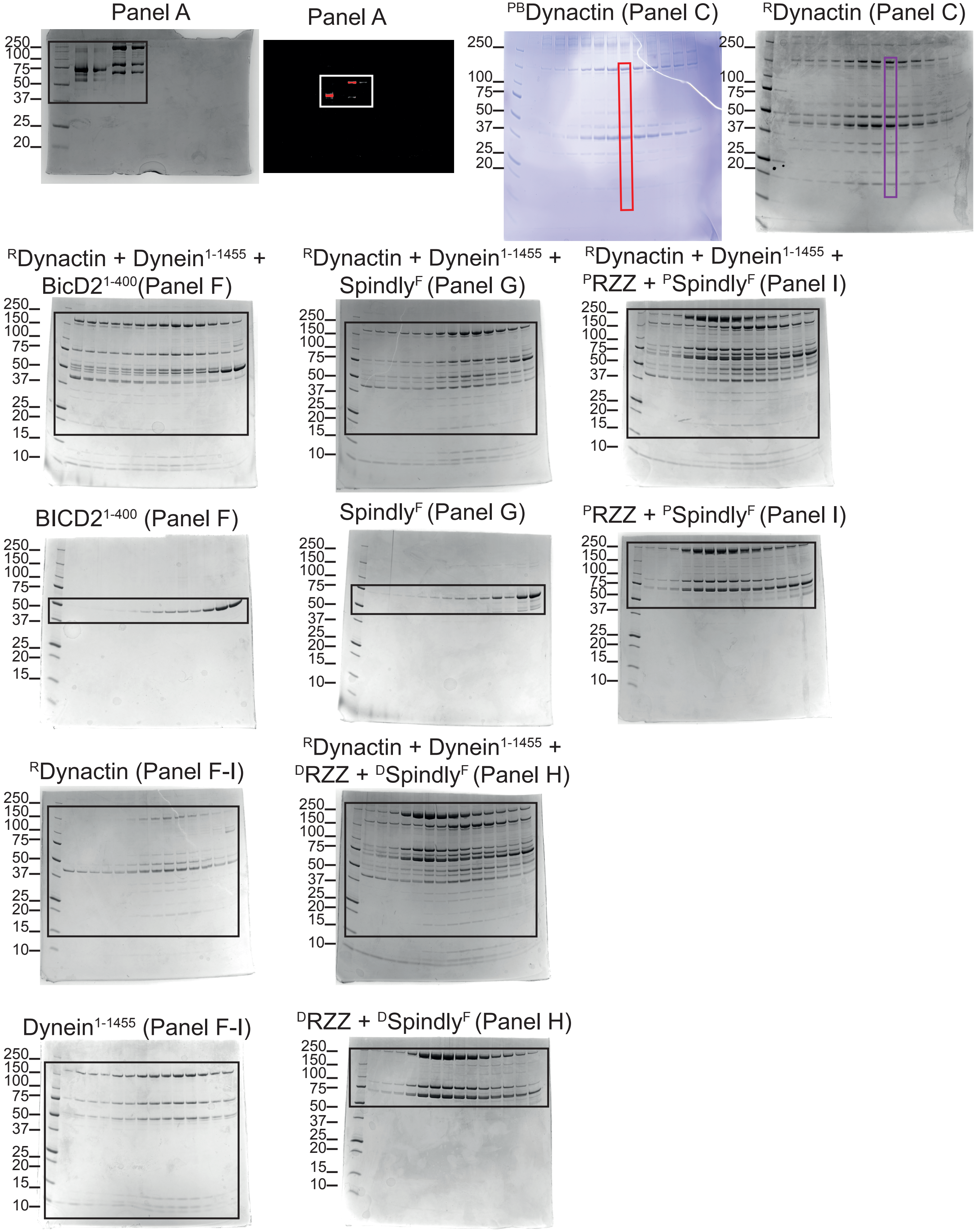

Panel A
250
100
75
50
37
25
20
Panel A
^{PB}Dynactin (Panel C)
250
100
75
50
37
25
20
^{R}Dynactin (Panel C)
250
100
75
50
37
25
20
^{R}Dynactin + Dynein$^{1-1455}$ + BicD2$^{1-400}$(Panel F)
250
150
100
75
50
37
25
20
15
10
^{R}Dynactin + Dynein$^{1-1455}$ + Spindly^{F} (Panel G)
250
150
100
75
50
37
25
20
15
10
^{R}Dynactin + Dynein$^{1-1455}$ + ^{P}RZZ + ^{P}Spindly^{F} (Panel I)
250
150
100
75
50
37
25
20
15
10
BICD2$^{1-400}$ (Panel F)
250
150
100
75
50
37
25
20
15
Spindly^{F} (Panel G)
250
150
100
75
50
37
25
20
15
10
^{P}RZZ + ^{P}Spindly^{F} (Panel I)
250
150
100
75
50
37
25
20
15
10
^{R}Dynactin (Panel F-I)
250
150
100
75
50
37
25
20
15
10
^{R}Dynactin + Dynein$^{1-1455}$ + ^{D}RZZ + ^{D}Spindly^{F} (Panel H)
250
150
100
75
50
37
25
20
15
10
Dynein$^{1-1455}$ (Panel F-I)
250
150
100
75
50
37
25
20
15
10
^{D}RZZ + ^{D}Spindly^{F} (Panel H)
250
150
100
75
50
37
25
20
15
10
