# Supplementary material for: Conformational transitions of the Spindly adaptor underlie its interaction with Dynein and Dynactin
Source: J Cell Biol. 2022 Sep 15;221(11):e202206131. doi: 10.1083/jcb.202206131 (PMC9481740; doi:10.1083/jcb.202206131)

Source Data FS7 B

IB: Spindly

mCherry FL Δ32 CC2* Chimera

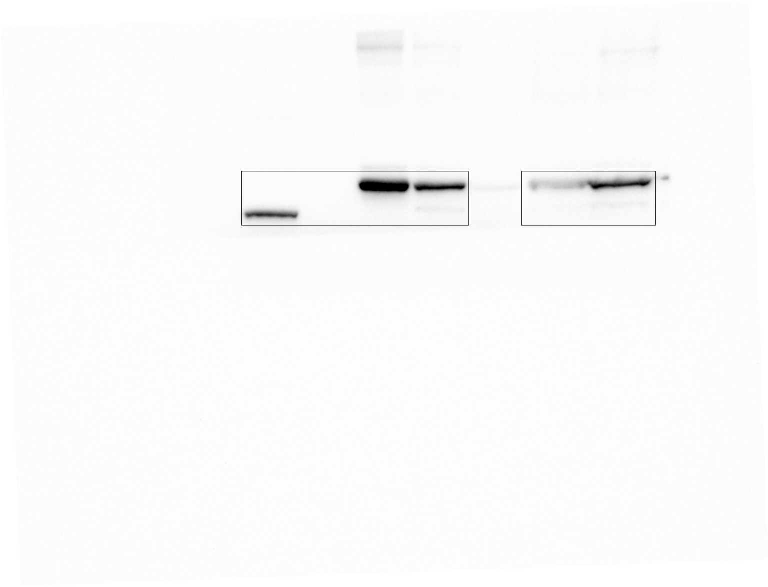

IB: Tubulin

mCherry FL Δ32 CC2* Chimera

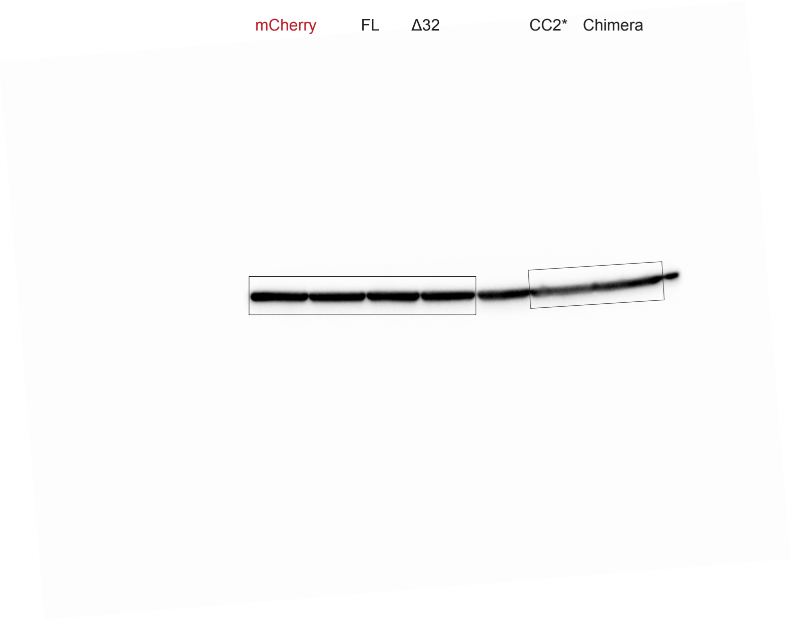

Merge of colorimetric + IB: Spindly

mCherry FL Δ32 CC2* Chimera

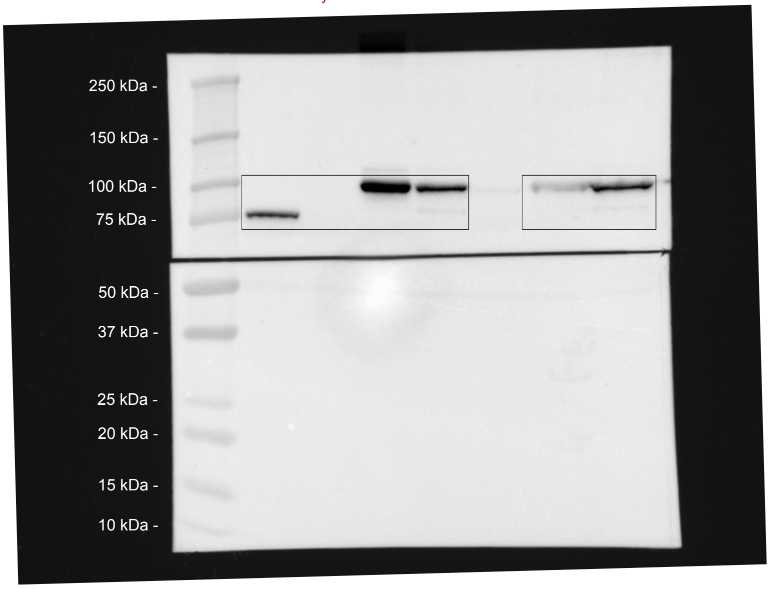

Merge of colorimetric + IB: Tubulin

mCherry FL Δ32 CC2* Chimera

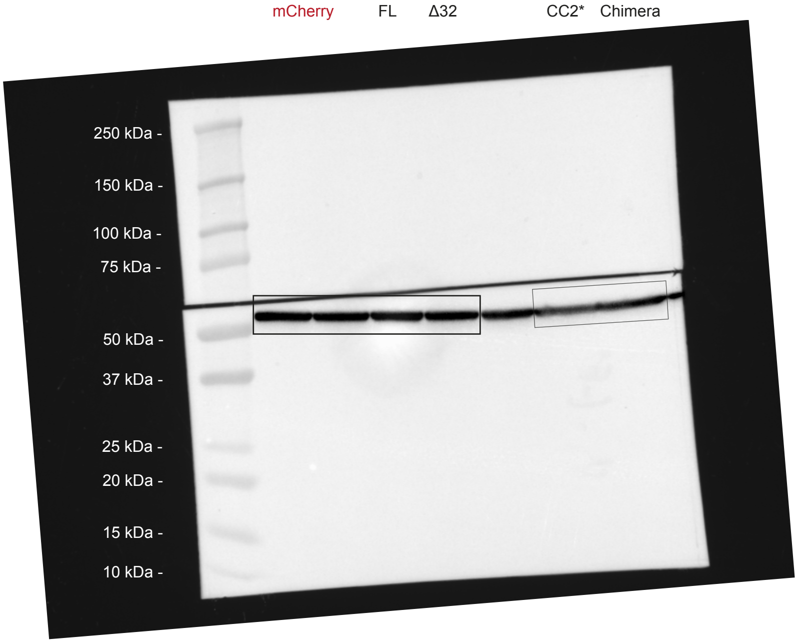

Supplement: SourceData FS7 — contains original blots for Fig. S7. [file JCB_202206131_SourceDataFS7.pdf]
